# The miR-200 Family: Versatile Players in Epithelial Ovarian Cancer

**DOI:** 10.3390/ijms160816833

**Published:** 2015-07-24

**Authors:** Goda G. Muralidhar, Maria V. Barbolina

**Affiliations:** Department of Biopharmaceutical Sciences, University of Illinois at Chicago, 833 S Wood Street, 335 College of Pharmacy Building, Chicago, IL 60612, USA; E-Mail: gmural2@uic.edu

**Keywords:** ovarian carcinoma, miR-200 family, metastasis, chemoresistance, expression

## Abstract

The role of microRNAs (miRNAs or miRs) in the pathology of epithelial ovarian cancer (EOC) has been extensively studied. Many miRNAs differentially expressed in EOC as compared to normal controls have been identified, prompting further inquiry into their role in the disease. miRNAs belonging to the miR-200 family have repeatedly surfaced over multiple profiling studies. In this review, we attempt to consolidate the data from different studies and highlight mechanisms by which these miRNAs influence progression of metastasis and chemo-resistance in EOC.

## 1. Introduction

The landmark studies of *lin-4* in *C. elegans* led to the discovery of a new class of molecules called microRNAs (miRNA, miR) [1,2]. According to the most recent data, 2588 mature human miRNAs have been identified and sequenced [3]. miRNAs are transcribed by RNA polymerases (II and rarely III) to form primary miRNA transcripts (pri-miRNA) [4]. The pri-miRNA is then enzymatically cleaved into pre-miRNA by Drosha and then exported to the cytoplasm. There, it is enzymatically cleaved by Dicer, leading to formation of the mature single-stranded miRNA [4]. MiRNAs bind to messenger RNAs as part of the RNA-induced silencing complex (RISC) and serve as post-transcriptional regulators of gene expression [5]. The seed sequences (nucleotides 2–8) of the mature miRNAs bind to the complementary region in the 3′UTR of mRNAs causing their degradation. Alternatively, when perfect complementarity cannot be achieved, or when miRNAs bind to the 5′UTR of the target genes, they inhibit translation [6,7].

Given the ability of miRNAs to control gene expression [5,8,9], they unsurprisingly became a focal point for their involvement in cancer. In fact, it has been found that miRNAs are frequently dysregulated in cancers [10,11,12,13] where they have been shown to contribute to pathogenesis, as well as disease progression and metastasis [14,15,16,17,18,19]. miRNAs may also serve as excellent surrogate markers for clinical response to drug treatments and outcomes [17,20,21]. Furthermore, oncogenic miRNAs that drive tumor progression could potentially be targeted for treatment [22,23,24,25]. Recent studies presented evidence that miRNAs can function as intercellular signaling molecules [26,27]. Consequently, we can glean from these findings that an effective miRNA signature for cancers would be of diagnostic, prognostic, and therapeutic value. This review focuses on epithelial ovarian cancer (EOC) in which the role of miRNAs has been extensively studied [20,28,29,30,31,32].

EOC is the leading cause of death from gynecologic malignancies and one of the deadliest cancers in women [33]. There are five different histotypes of EOC: high grade serous, low grade serous, endometrial, clear cell, and mucinous [34]. Each of the histotypes has been found to be associated with mutations in specific genes and have different clinical manifestations [35]. Most of the patients are diagnosed at late metastatic stages when there is minimal chance for survival due to the lack of effective anti-metastatic treatments. Incidence of ovarian cancer has been steadily increasing over the past century, while development of more effective treatment options has lagged behind, resulting in no improvement in overall survival. While current standard of care, a combination of surgery and chemotherapy, is efficient as initial treatment, in most cases EOC recurs after a few years and becomes resistant to existing treatments [36,37]. Inability to prolong patient remission is a critical gap in the clinical management of EOC. The underlying cause of this problem stems in part from insufficient basic knowledge of the biology and mechanisms supporting EOC metastasis.

Owing to their versatile functions, miRNAs can be instrumental in improving our understanding and treatment of EOC [32]. Many miRNAs have been found to be differentially expressed in ovarian carcinomas compared to normal tissues. Due to the high frequency of genomic alterations in miRNA genes in ovarian cancer, a corresponding degree of miRNA dysregulation has also been observed [38,39]. The dysregulated miRNAs in ovarian cancer, as well as their clinical significance, has been reviewed elsewhere [32]. Recent analysis of the Cancer Genome Atlas (TCGA) data identified a gene network along with the predicted regulatory miRNAs that characterized a pro-malignant mesenchymal phenotype of serous EOC [40]. They showed that 89% of the target genes in the network were regulated by 8 key miRNAs. Two of these key miRNAs, miR-141 and miR-200a, are members of the miR-200 family.

**Figure 1:**
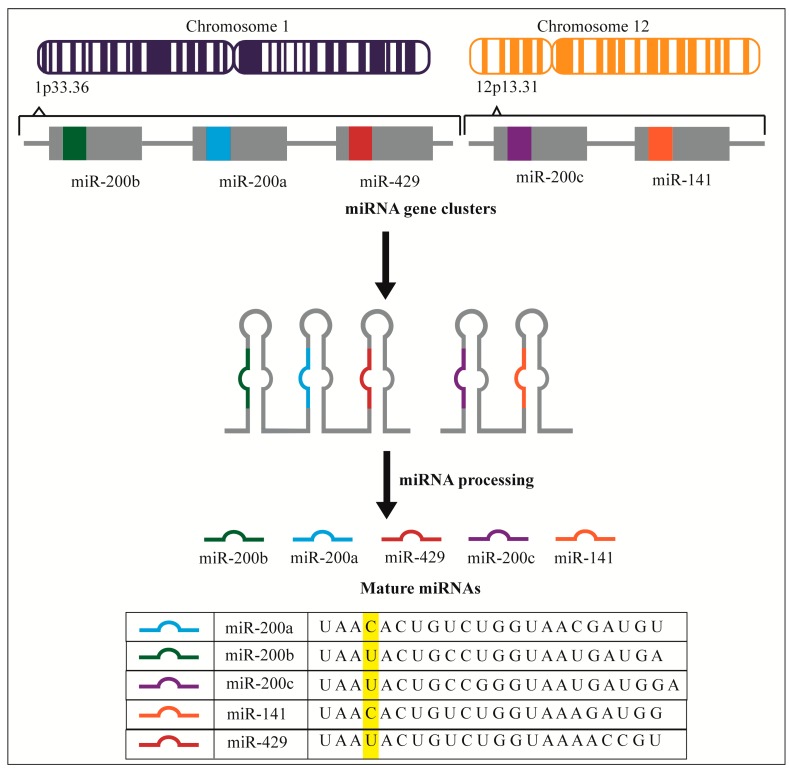
miRNA-200 family arises from two gene clusters: miR-200b, miR-200a and miR-429 from chromosome 1 (1p33.36) while miR-200c and miR-141 from chromosome 2 (12p13.31). The highlight indicates that in the seed sequence (nucleotides 2–8) the difference is only in one nucleotide.

The miRNA-200 family (miR-200 family or miR-200) has repeatedly been implicated for its involvement in EOC as well as other cancers [41,42,43]. This family consists of miR-200a, 200b, 200c, 141 and 429 (Figure 1). They arise from two different gene clusters: miRs-(200a/200b/429) from chromosome 1 (1p36.33) and miRs-(200c/141) from chromosome 12 (12p13.31). They share a high degree of sequence homology with a difference of only one nucleotide in their seed sequence (nucleotides 2–8) and regulate expression of many of the same target genes. Here, we present a review of the current scientific literature on the expression and role of the miR-200 family in EOC.

## 2. miR-200 Expression Profiles

One approach to understanding the impact that miRNAs have on cancers is to identify the miRs that are aberrantly expressed in them. With the advent of superior profiling technologies, multiple studies were performed in order to identify the miRNAs that were differentially expressed in EOC and could be linked to pathogenesis and disease progression. Across multiple studies with different detection platforms and over extensive sets of tumor tissues, cell lines, and large sets of normal control samples, differential expression of the members of the miRNA-200 family is a consistent finding. These studies have been summarized in Table 1.

A comparative genomic hybridization study of epithelial cancers, including ovarian cancer, showed frequent alteration in loci containing miRNA genes resulting in aberrant miRNA expression profiles [38]. Specifically, the miRNA-200 family genes showed copy number gains. This indicated an increased expression of miRNA-200 which was also confirmed by another study that compared expression profiles of normal ovarian tissue and ovarian cancer to determine a miRNA signature for ovarian cancer [44]. The major finding was the up-regulation of miR-200a, 141, 200c and 200b. Moreover, miR-200a and miR-200c showed increased expression in serous, endometrioid and clear cell cancer while miR-200b and miR-141 were up-regulated in endometrioid and serous histotypes thereby indicating histotype specificity. Another study with a smaller number of samples also showed that miR-200a expression was increased in ovarian tumor tissues [45]. Their results showed that miR-200a overexpression along with miR-199a* and miR-204 was associated with high grade and late stage tumors thus suggesting a role in tumor progression. These studies establish a compelling argument for elevated miR-200 family expression as a significant characteristic of ovarian tumors compared to their non-neoplastic counterparts.

**Table 1:** miRNA 200 family expression in ovarian cancer profiling studies.

Study	Samples & Normal Controls	miRNA 200 Family Expression	Conclusions Made by Authors
Iorio *et al.* [44]; Ohio State Comprehensive Cancer Center microarray, version 2.0 with 460 mature miRNA probes (235 human miRNAs)	Samples: 69 malignant tumor tissues (including serous, endometrioid, clear cell, poorly differentiated and mucinous ovarian carcinoma); Controls: 15 normal ovarian tissue sections	Increased expression of miR-200a, 200b, 200c and 141 in tumor samples *vs.* normal tissue	MiR-200a, 200b, 200c, and 141 share a common putative target BAP1 (BRCA associated protein 1), a tumor suppressor down-regulated in ovarian cancer
Yang *et al.* [45]; Oligonucleotide array, GeneScreen Plus (NEN) membranes printed with 515 human and mouse miRNA probes	Samples: 10 human ovarian epithelial tumors; Controls: Normal ovarian tissue and immortalized human ovarian surface epithelium	43% of primary ovarian carcinomas showed increased miR-200a expression	Increased miR-200a expression was associated with high grade and late stage disease
Dahiya *et al.* [46]; miRCURY™ LNA miRNA arrays with 1458 probes for all miRNAs in miRBase Release 8.1 (Exiqon)	Samples: 34 cancer tissues and 10 ovarian cancer cell lines (BG-1, UCI-101, HEY, OVCA420, OVCA432, OVCA433, OVCAR2, OVCAR3, OVCAR5, OV90); Controls: HOSE-B cells (human ovarian surface epithelial cell line immortalized with E6 and E7)	MiR-200a and 141 were found to be down-regulated in the neoplastic samples	Using Target Scan 3.0 miR-200a and 141 were found to share three predicted targets (ZEB2, KLF12 and ZFR)
Wyman *et al.* [47]; Parallel pyrosequencing (454 Life Sciences Platform)	Samples: Stage III/IV ovarian tumors including 19 serous, 4 clear cell and 10 endometrioid; Controls: 4 Normal primary human ovarian surface epithelium (HOSE) and E6/E7 immortalized HOSE	MiR-200a, 200b, 200c, 141, and 429 showed increased expression in ovarian tumors and the immortalized HOSE	Normal HOSE expresses low levels of miR-200 family. Immortalization induces their expression
Lee *et al.* [48]; Microarray with 668 Ambion probes (328 known and 154 predicted human miRNA probes)	Samples: 37 serous tumors (including high grade, low grade and borderline serous tumors); Controls: 3 normal fallopian tube epithelium sampled from the fimbriae	In high grade serous tumors miR-200c and 141 were up-regulated; In low grade serous tumors, miR-200a, 200b, 200c, and 141 were up-regulated	MiR-200a, 200b, 200c, and 141 were up-regulated in serous tumors. This was the first study that used fallopian tube epithelium as normal control as opposed to ovarian surface epithelium
Bendoraite *et al.* [49]; qRT-PCR using Taqman miRNA assays (Applied Biosciences)	Samples: Stage III/IV malignant ovarian primary tumors from 70 patients (including serous, endometrioid, and clear cell histotypes), 15 ovarian cancer cell lines (A1847, A2780, CaOV3, ES-2, HEY, IGROV1, OVCAR3, OVCAR5, OVCAR10, OV-90, PEO-1, SKOV3, TOV-21G, TOV-112D, 2008); Controls: Non-immortalized early passage primary cell cultures derived from HOSE as normal controls	Expression of all five members of miR-200 family were substantially higher in the primary tumors compared to normal tissues	Low expression of ZEB2 and high expression of miR-200 family in the tumor samples supports mesothelial to epithelial transition model

The cell of origin in ovarian cancer has been debated [50,51,52,53]. This adds to the complexity of interpreting the data from the profiling studies as the results may differ based on what cells are being used as “normal” controls [54,55]. In the profiling studies undertaken so far, the whole ovary, ovarian surface epithelium (both primary and immortalized), and fallopian tube epithelium have been used as controls. miRNA profiling of serous ovarian cancers compared to fallopian tube epithelial cells showed that miR-200a, 200b, 200c and 141 were up-regulated in low grade serous cancer whereas only miR-200c and 141 were up-regulated in high grade serous tumors [48].

A recent study that utilized parallel pyrosequencing, compared miR expression in normal human ovarian surface epithelium (HOSE) cells and immortalized HOSE cells to that in late stage ovarian tumors [47]. It was found that the miR-200 family expression increased in the HOSE cells following immortalization using E6/E7 viral proteins [47]. This indicated an increase in miR-200 expression in cancer cells while compared to HOSE cells, and this result would have been missed when compared to immortalized HOSE cells [46].

Finally, the data from the cancer genome atlas study showed a down-regulation of miR-200a in the mesenchymal subtype of serous ovarian cancers [40]. These differences in results can also potentially indicate a mechanistic alteration in expression depending on various factors among which could be the disease stage, the histotype, and whether or not the tumor is metastatic. Information regarding the mechanism of action of miR-200 suggests that the key may lie there.

Following the discovery of the presence of miRNAs in exosomes [56], tumor-derived exosomes became possible surrogate markers for diagnosis, prognosis, and clinical outcomes [57]. Interestingly, exosomes derived from the peripheral circulation of patients with ovarian tumors displayed a similar expression pattern of the miR-200 family as the tumor cells [58]. Elevated levels of miR-200a, 200b and 200c were also observed in the serum of serous ovarian cancer patients [59].

## 3. miR-200 and Metastasis

There is a very high degree of sequence homology between the members of the miR-200 family [60] (see Figure 1). Due to the homology in their seed sequences, they share several targets. Two established targets of miR-200 are the zinc finger transcriptional repressors: ZEB1 (TCF8/ZFHX1A/δEF1) and ZEB2 (SIP1/ZFHX1B/SMAD1P1) [40,61,62,63,64,65]. The ZEB transcription factors bind to the E-boxes in promoter regions of E-cadherin and cause transcriptional repression of E-cadherin expression [66,67,68,69]. E-cadherin is a critical protein for maintenance of the epithelial phenotype. ZEB-mediated loss of E-cadherin causes cells to develop spindle-shaped morphology and express greater migratory and invasive potential [70]. The ability to manipulate ZEB expression makes miR-200 ideally positioned to influence the process of epithelial to mesenchymal transition (EMT) [42,71,72]. Further, there exists a double negative feedback loop between miR-200 and the ZEB genes [73,74]. ZEB binds to E-boxes in the miR-200 promoter and thereby suppress their expression. While miR-200 causes post-transcriptional repression of ZEB, the latter regulates transcriptional repression of the miRNAs [75]. This double negative feedback loop allows greater flexibility over cell fate, but complicates attempts to understand the reversible EMT process, especially in terms of isolating the initiating events. Also, some degree of variation in the effect on EMT has been reported between the different members of the miR-200 family [76]. There has been evidence supporting a mesothelial to epithelial transition (MET) in normal cells during ovarian tumorigenesis that involves increased miR-200 expression [49]. The ovarian cancer cells could later undergo the traditional EMT during metastatic dissemination. This raises the possibility of a dual expression profile of miR-200 during tumor progression [49].

It has been shown that TGF-β mediated down-regulation of miR-200 in mesothelial cells promotes cancer cell attachment and proliferation [77]. Additionally, reduced miR-200 expression causes increased activity of its targets, Interleukin-8 and chemokine ligand CXCL1, secreted by both endothelial as well as tumor cells [78] resulting in increased angiogenesis and metastasis. Both studies demonstrated that delivery of miR-200 in mouse models as therapy caused suppression of metastatic dissemination. Even as more information regarding the potential of miRNA-based therapeutics is gathered [79], development of successful miRNA delivery systems remains a challenge [80,81]. Yet, in light of their involvement in ovarian carcinoma metastasis, a miR-200-based therapeutic strategy [82] could prove to be promising.

## 4. Effect on Chemotherapeutic Response and Clinical Outcomes

Current treatment options for ovarian cancer include surgical resection followed by chemotherapy. The drugs used for first line therapy include a combination of carboplatin (a platinum-containing alkylating agent) and paclitaxel (a microtubule-targeting agent). Since most cases are diagnosed at a late stage, the high rate of response of stage I patients to therapy is overshadowed by the relapse and mortality of patients diagnosed late. The relapse of the cancer is mediated by loss of sensitivity to the chemotherapeutic agents. Unsurprisingly, miRNAs seem to be significant players in therapy resistance [29].

Alteration in the expression of class III β-Tubulin (TUBB3) is one of the mechanisms by which ovarian cancer cells gain resistance to microtubule-targeting agents [83,84,85]. It has been shown that miR-200c binds to the 3′UTR of TUBB3 and down-regulates its expression thereby robustly sensitizing the cells to paclitaxel as well as other microtubule targeting agents, such as vincristine and epothilone [86,87]. In a follow-up study, all the other members of the miR-200 family were also shown to be regulating TUBB3 levels [88]. In addition, low expression of miR-200 was shown to be a marker for poor survival and resistance to paclitaxel in ovarian cancer patients [88,89]. However, further investigation of the interaction of miR-200c and TUBB3 along with the involvement of an RNA Binding Protein-HuR exposed the complexity of the underlying mechanism. It has been shown that cytoplasmic HuR causes stabilization and increases the levels of TUBB3 [90] in conjunction with miR-200c [91] leading to poor survival. This is in direct contrast to the previous findings and prompted the researchers to propose a model that describes the two different mechanisms by which miR-200c regulates *TUBB3* mRNA in ovarian cancer. According to this model, when HuR is located in the nucleus, high levels of miR-200c are favorable and cause down-regulation of TUBB3. On the other hand, cytoplasmic HuR causes a miR-200c-mediated increase in TUBB3 leading to paclitaxel resistance and poor outcomes [91]. This could also potentially explain the recent findings that showed high miR-200a, 200b and 200c expression correlated with poor overall survival [92].

Studies performed in mouse models showed that increased expression of miR-141 and miR-200a increased tumor growth [93]. However, the miRNAs were also responsible for repressing p38α that produces oxidative stress response, which was shown to improve clinical outcomes [93]. Reactive oxygen species (ROS) have been shown to play a crucial role in sensitizing cells to paclitaxel treatment, and the cells producing the oxidative stress response showed better response to paclitaxel [94,95]. This paradox led the investigators to propose a model that explains the cross-talk between miR-200, p38α, and ROS. In normal cells, there is a balance between these players [93]. In a neoplastic cell that is still in the early stages of transformation, there is an increased concentration of ROS prompting up-regulation of the miRNAs, which in turn represses p38α. These conditions produce a state of oxidative stress, which improves sensitivity to paclitaxel. As the tumor progresses, down-regulation of the miRNAs restores p38α expression thereby causing cells to become resistant to paclitaxel.

The miR-200 family has also been identified in ovarian cancer survival and clinical outcomes studies [88,89,96,97,98]. It was shown that higher expression of miR-200a, 200b, 200c and 141 was part of a miRNA signature that significantly correlated with decreased progression-free survival and overall survival in ovarian cancer [92,96]. Conversely, results from other studies showed that higher expression of miR-200a was predictive of better outcomes and survival in ovarian cancer [78,97,98] and that the expression decreased with stage [98]. Similar results with the associations between miR-200c with overall survival and progression-free survival have also been shown [89,99]. These discrepancies in the data from previous findings were suggested to be related to differences in profiling platforms [97] and insufficient staging information at the time of diagnosis [98]. In a large study, serum from 74 ovarian cancer patients, 19 borderline patients and 50 healthy controls were extracted and the levels of miR-141 and 200c were measured. While their elevated expression could be used to distinguish patient from healthy controls, higher expression also correlated with increased survival [100]. Some of the studies that investigated the relationship between miR-200 and clinical outcomes have been summarized in Table 2.

**Table 2:** Predictive value of miRNA-200 family expression for disease outcomes.

Study	Samples	miRNA 200 Family Expression	Conclusions Made by Authors
Nam *et al.* [96]; Microarray with 377 (314 human) mirVana miRNA probes (Ambion)	Samples: 20 serous ovarian cancer tissues: 9 chemo-resistant, 11 chemo-sensitive tumors; Controls: 8 normal ovarian tissues	Increased expression of miR-200a, 200b, 200c and 141 in tumor samples *vs.* normal tissue	High expression of miR-200a, 200b, 200c and 141 were significantly correlated with decreased progression-free survival as well as overall survival
Hu *et al.* [97]; qRT-PCR miRNA assays (Applied Biosystems)	55 patients: 48 epithelial ovarian carcinomas and 7 primary peritoneal carcinomas	Disease recurrence and poor overall survival were associated with low miR-200a, 200b and 429 expression	miR-200b-429 cluster expression has prognostic value in EOC
Eitan *et al.* [98]; Custom microarray slide (Nexetrion^®^) with 900 miRNA probes	57 patients who had undergone surgery for tumor resection: 19 Stage I patients, 38 Stage III patients; All received platinum based chemotherapy	miR-200a expression was higher in Stage I ovarian cancer compared to Stage II	The data set shows significantly higher expression of miR-200a in early stage disease correlating with improved survival
Marchini *et al.* [89]; G4470B human miRNA microarray (Agilent Technologies) with probes for 723 human miRNAs	144 patients with Stage I EOC out of which 29 patients relapsed	Tumors with lower miR-200c levels seen in patients who relapsed	miR-200 expression could be used as an indication of relapse in Stage I tumors
Leskela *et al.* [88]; qRT-PCR using the miRCURY™ LNA miRNA assay kits (Exiqon)	72 patients were studied for overall survival analysis; A subgroup of 57 patients with both advanced tumor stage and serous carcinoma histotype were studied for treatment response	miR-200 expression correlated with β-Tubulin III levels	Low miR-200 expression was seen in patients without complete response to paclitaxel when compared to patients with complete response; Low miR-200 expression had a trend towards poor survival

## 5. Conclusions

**Figure 2:**
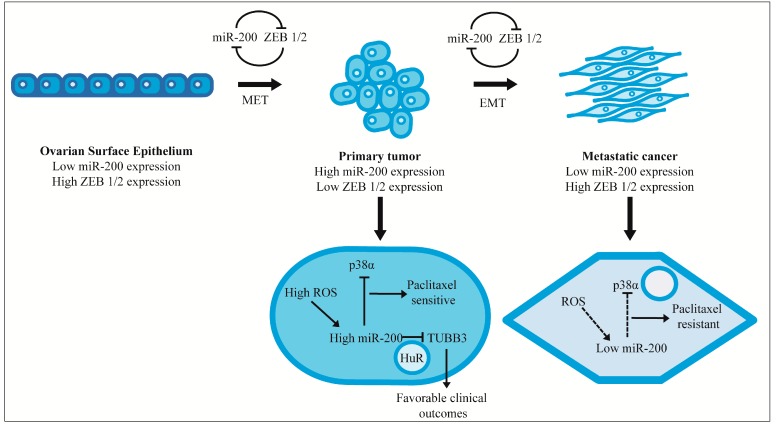
Model for the expression and mechanisms of action of miR-200 adapted from Bendoraite *et al.* [49], Mateescu *et al.* [93] and Prislei *et al.* [91]. miR-200 could regulate tumorigenic and metastatic transformation by Mesothelial to Epithelial Transition (MET) and Epithelial to Mesenchymal Transition (EMT) respectively. miR-200 expression aided by ROS represses p38α and increases sensitivity to paclitaxel. In cancer cells with low miR-200 expression, this process is not active leading to paclitaxel resistance. Another mechanism involved is the miR-200 mediated down-regulation of TUBB3 in cells with nuclear HuR leading to better clinical response and treatment outcomes.

In spite of such extensive investigation, the expression and role of miR-200 in EOC remains a point of contention. Available data indicate that these miRNAs are subject to dynamic changes depending on the stage of tumor progression, EMT, nuclear or cytoplasmic localization of interacting proteins and the cellular ROS content; it will, to an extent, explain the discordant data in the profiling studies. A compilation of some of the published findings pertaining to miR-200 [49,91,93] lends itself to a possible model of tumor progression in ovarian cancer as shown in Figure 2. Depending on where and when the tumors are sampled from, they may exhibit very contradictory expression patterns. It might be beneficial to perform preliminary studies in animal models in order to standardize the normal cell controls, tumor stage, site and histotype among other variables. Effectively establishing the role of miR-200 in EMT and chemo-resistance will hopefully open new avenues for therapeutic intervention. Irrespective of the exact associations, it is quite clear that these miRNAs are indeed versatile players in the EOC microenvironment.
